# The toxicology and detoxification of *Aconitum*: traditional and modern views

**DOI:** 10.1186/s13020-021-00472-9

**Published:** 2021-07-27

**Authors:** Yau-Tuen Chan, Ning Wang, Yibin Feng

**Affiliations:** School of Chinese Medicine, The University of Hong Kong, Hong Kong, China

**Keywords:** *Aconitum*, Aconitine, Toxicology, Drug processing, Detoxification

## Abstract

*Aconitum carmichaeli Debx.-*derived herbal medicine has been used for anti-inflammation and anti-arrhythmia purpose for more than two thousand years. It is processed into *Chuanwu* (*Radix Aconiti praeparata*) and *Fuzi *(*Radix Aconiti lateralis praeparata*) in Traditional Chinese Medicine, which are two useful drugs but with toxic properties. There have been patients poisoned by accidental ingestion of *Aconitum* plants or misuse of the herbal drug, and this is of great concern to study in-depth. In this review, we provided the traditional and contemporary practice of using *Aconitum* herbs as medicine, from functions, processing methods to toxicity in ethnomedicine aspects to discuss the underlying connections of traditional and modern understanding on the toxicity of *Aconitum* plants. We summarized the functions and toxicology of the herbal drugs are analyzed from chemical and clinical aspects, with the help of traditional and modern knowledge of medicine. The medicinal doses and lethal doses determined by researches are summarized, and the usage and processing methods are updated and reviewed in the modern view. In addition, clinical management of poisoned cases using western medicine is discussed. This review provides insights and awareness of safety when using *Aconitum*-derived herbal medicine, and the application of modern scientific knowledge to optimize the detoxification processes. We suggest the possibility to renew the current standard processing method from the official Pharmacopoeia all over the world*.*

## Introduction

*Aconitum* is a genus of herbal medicine in the *Ranunculaceae* family, with more than 400 species all over the world [1]. *Aconitum* plants, also having the names of aconite, monkshood, wolf’s bane, queen of poisons, are a branch of herbal drugs in traditional Chinese Medicine [2]. *Caowu*, *Chuanwu, Fuzi* and *Tianxiong* are four examples of Chinese herbal drugs that are deriving from the same species *Aconitum carmichaelii* Debx., which is the common herb to be used nowadays [3]. The medical use of *Aconitums*, regardless of the raw or processed herbs, are highly cautious since its obvious toxicity. Despite the potential danger, the clinical applications of *Aconitum* species are becoming more systematic with the increasing understanding of its toxicology and methods of detoxifications.

There have been poisoned cases reported even until recent years in herbal medicines used regions, including China, Hong Kong [4], Taiwan and India [5], etc. It may sound dangerous to use these kinds of toxic drugs. However, due to the essential role of the *Aconitum* in many diseases, including rheumatism, joint pains [6], oedema, gastroenteritis [7], asthma, abdominal pains [8, 9], and some gynaecological disorders like irregular menstruation and dysmenorrhea, it is a very efficient herbal drug to ease pains, as a result of its effect on the neuronal cells, which will be described in detail later. It is a restricted Chinese medical drug to be used, but not prohibited in Hong Kong. Therefore, it is important to investigate in-depth the effect, toxicity, risk, and treatment related to the toxification of the *Aconitums*. In this review, we retrieved literature from the PubMed database and summarized the traditional use and the recent advances in the investigation of toxicity, toxicology and processing to detoxification of *Aconitums*. With a clinical-oriented aspect, we also focus on the clinical symptoms and management of *Aconitums* intoxication in humans. The toxicokinetic information of *Aconitum* alkaloids is investigated by Yang et al*.* in the recent article [10], and that would not be covered in this review.

## Ethnophamracological relevance of Acontitum toxicity

### The use of Aconitums in traditional medicine

China has a long history of using *Aconitum* as a herbal drug. It was first recorded in *Shennong’s Materia Medica*, the very first Chinese herbal medicinal classic dated around two thousand years ago. More detailed information, taxonomy, usage on the species *Aconitum carmichaeli Debx.* derivatives *Chuanwu* (*Radix Aconiti praeparata*) and *Fuzi (Radix Aconiti lateralis praeparata)* were illustrated in another classic “*Shanghan Lun”* written by the master Zhang Zhongjing in the Eastern Han Dynasty [11]. It was classified as a “lower-class” drug, and marked as “very poisonous” that must be used with extreme care. The *Aconitum* species were categorized as the “warm” drugs, that can power up and energize the body, dispel moisture and humidity, as well as ease pain. *Fuzi* was included in more than 20 herbal drug formulae [12]. The amounts of *Chuanwu* and *Fuzi* that could be prescribed to a patient were listed in the traditional *materia medica.* For *Chuanwu,* the amount should be 1.5 ~ 3 g (translated to Standard Unit) in formulation, while used as a single drug should not be more than 2 g. The amount of *Fuzi* could be as high as 15 g, as the toxicity of *Fuzi* is less toxic, especially in the processed form. In recent years, prescribing *Fuzi* becomes one of the most crucial schools of thought in Chinese medicine.

Apart from the physicians from the past, *Aconitum* herbs were also used locally as folk medicine or supplement. People from the rural villages in the Qinling Range of the Shaanxi Province in China used to cook and consume *A. carmichaeli* before winter [13]. They had a saying that taking the herbs will give them warmth during the winter, and also energy for everyday work. They would not cook the herb like other vegetables, but cutting the root into slices, and boiling in a soup. The soup was kept boiling to dry up and added water back several times. The whole process should last for hours to several days. They claimed very few people felt unwell after consuming *Aconitum* in this way.

Other than the ancient Chinese, Indian also has a long history in alternative medicine, or non-allopathic medicine [14, 15]. There were different systems of medicinal practice including Unani, Siddha, Ayurveda, etc., but would not be discussed in detail here. They have *Aconitum* herbs introduced as drugs, and they also realized the toxicity of aconitine (AC). Before the usage of the herb, the Indian would boil the root of the plants with cow’s urine for two days. It is then washed with water and boiled again with milk for two more days. After that, the residue will be cut and dried. They preserved the product in powdered form. The Indian used *Aconitum* herbs to combat fever, inflammation, emesis and diarrhoea [16]. However, toxic injured and fatal cases are reported. It is observed that boiling for a prolonged time is a rural method of processing the *Aconitum* herb to reduce toxicity.

### Traditional processing methods of Aconitum in TCM

*Paozhi* is the Chinese term for processing crude herbal drugs using specific methods, that can reduce the toxicity of the drug while maximizing its effectiveness. There can be more than one method to process the same herb, that can bring about different pharmacological functions [17].

**Fig. 1:**
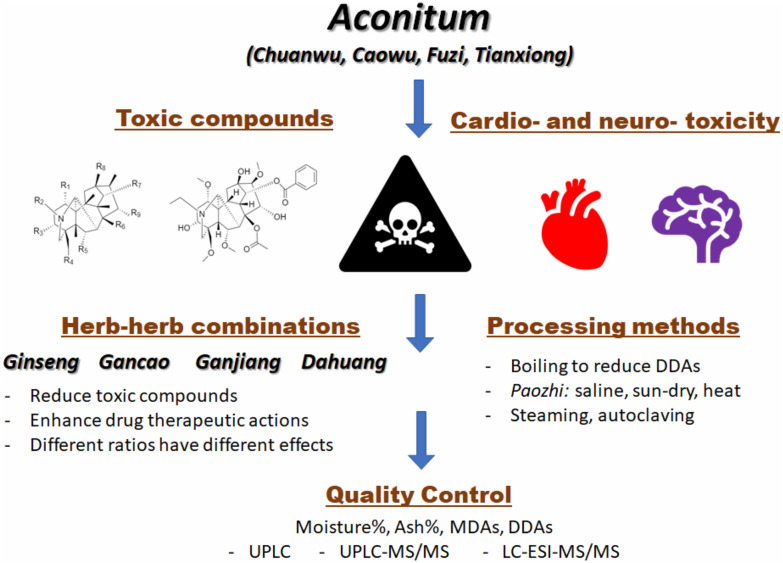
Graphical abstract of the *Aconitum* toxicology and detoxification

In this review, only the processing methods of *Aconitum carmichaeli* Debx. herb would be reviewed (Fig. 1). The raw plant *Aconitum carmichaeli* are commonly cultivated in the Sichuan Province, but also natively found in Russia, Japan and other East Asia region [18]. It is a perennial plant that has a purple in colour flower which could be 60–150 cm tall when mature completely. This plant is usually grown on a grass slope, in between bushes. The underground tuber root should be collected in early summer, from June to August, just before the flowering period. This plant could be derived into three different types of herbal drugs, namely “*Shengchuanwu*”, “Zhichuanwu” as well as “*Fuzi*” [19, 20], according to the Pharmacopoeia of the People’s Republic of China, 2020.

“*Shengchuanwu*” is derived from the mother root of the radix plant by drying, “*Zhichuanwu*” is the processed form of “*Shengchuanwu”*, while “*Fuzi*” is processed from the daughter root of the same plant. “*Shengchuanwu*” is produced rather simple by drying under the sun after the lateral roots, sand and soil are removed. When tasted on the tongue, one should feel slight numbness. From the raw “Shengchuanwu”, the large root part is soaked into water, then boiled for 4–6 h or steamed for 6–8 h, and then sliced and dried under the sun. This processed form of *Chuanwu* is the *“Zhichuanwu.* On the other hand, “*Fuzi*” and its derivatives are much more complicated. “*Fuzi”* means *attached offspring* in Chinese, which is the daughter root of the radix. *Fuzi* itself are poisonous as well, therefore it must be processed before use. The crude *Fuzi*, which is also called “*Nifuzi*” or “*Zhifuzi”*, is selected lateral roots with soil and sands removed. It could be further processed into five different herbal drugs. “*Yanfuzi”* is made by soaking *Nifuzi* into saline with repetitive drying until it is hardened and the surface is coated with salt crystals. “*Heishunpian”* is another type of processed *Fuzi*, which in addition to soaking in saline, it is also boiled and stained to become dark, or black in colour. They should be dried under the sun and will have no numbness feels when tasted. The third one is “*Baifupian”,* which are produced using the root with the bark skin removed. They are boiled in saline as well and dried under the sun. To enhance its white colour, sometimes the product will be steamed with sulphur. The fourth one is “*Danfupian*”, which is made by washing the *Yanfuzi* in water until the salt is complete removed, and boiled with Licorice and black beans. The final one is “*Paofupian*”, which is *Fuzi* processed by deep frying in hot sands until plump and slightly coloured.

There are some other *Aconitum* species also used for herbal drug, including *Aconitum kusnezoffii* Radix *(Caowu)*, *Aconitum kusnezoffii* Radix cocta. *(Zhicaowu)*, *Aconitum kusnezoffii* Folium *(Caowuye)*, *Aconitum coreanum* Rapaics *(Guanbaifu)* and *Aconitum pendulum* Busch *(Xueshangyizhihao).*

## Medicinal effects of *Aconitum* species

In TCM practice, herbal drugs are given to patients in a compound formulation, instead of a single drug or compound. According to the TCM theory, most of diseases are induced by an imbalance of the inner “*yin-yang*” in the human body. Different diseases are the results of disturbance of different organ system, and so prescriptions are usually given in a formulation. The complex compounds present in the formulation could help the body regain its original optimal state.

### Effects in the cardiac system

In the school of Chinese Medicine, “*huoshen*”, which prefers to use *Aconitum* a lot in many decoctions, this herb is important to enhance cardiopulmonary functions. Other than the toxic effect mentioned previously, as a drug, the *Aconitum* herbs also bring beneficial effects to the cardiac system [21]. As recorded in the Chinese Classics, the *Fuzi* has the effect of “warming up and energize” bodies. This could be described in modern medicine using the cardiotonic actin [22]. The major energy source of the cardiac muscle is adenosine triphosphate, which is produced mainly from cellular respiration in the mitochondria, and a minority of them from glycolysis [23]. Adenosine monophosphate-activated protein kinase (AMPK) is an enzyme that regulates cellular energy homeostasis by activating glycolytic and fatty acid metabolism [24]. Deng et al*.* showed that Benzoylaconine (BAC) could promote mitochondria accumulation and ATP production in a dose-dependent manner. BAC could activate oxidative phosphorylation related protein expression in various organs including the heart, and increase oxygen consumption. Other compounds are also involved, including napelline, lappaconitine (LA) and 6-benzoylheteratisine [25]. LA, and its metabolite *N*-deacetyllappaconitine (DLA), showed antiarrhythmic actions while having a lower toxicity. Mice have 500 to 100 times higher tolerance in terms of causing arrhythmia when comparing LA and DLA to AC [26].

*Shenfu Tang, Shenfu San, or Shenfu Injection* (SFI), are a series of herbal drug formulas that involve the usage of *Fuzi*, as well as *Ginseng (Radix ginseng)* [27]. Both of these drugs have very effective power in energizing severe patients and help recover from chronic illness. An example was to be applied to the treatment of anaemia in patients with lung cancer. After 14 days of intravenous injection of SFI, the performance status of the patients was generally improved when compared with the control group, measured using the Karnofsky score. In the meantime, the anaemia situation and immune level were also improved [28]. It was also reported that SFI (~ 10 mg/24 h, i.v.) had positive effects in the recovery of patients suffering from myocardial infarction [29]. Sixty myocardial infarction patients were given the cardiogenic shock and treated with an intra-aortic balloon pump, and then half of them were given SFI. The result showed that SFI significantly shortens the recovery time of the patients, by reducing the inflammation response.

### Analgesic effects

In TCM, processed *Aconitum* or *Fuzi* are used as an analgesic agent when treating diseases like rheumatoid arthritis [30]. It is shown that the alkaloids, other than the toxic ones, in the plant have a different extent of analgesic activity [31–33]. Researches indicated that AC, as well as 3-acetylaconitine and hypaconitine (HA), were the highest affinity to the sodium channel. When AC molecules bind to the sodium channel, at site II, the ionic flow through the channel will decrease. In that case, the sodium ion influx is affected, which changes the selectivity of the channel. The opening of the sodium channel is prolonged, which results in an extension, or even permanent depolarization of neurons. This corresponds to blocking of synapse signally passage, and thus account for the antinociceptive and analgesic effect of AC [34]. This was found in the hippocampus, where neuronal conduction was inhibited. Nevertheless, these three compounds are also having high toxicity. The ratio of ED_30_ to analgesia is similar to the LD_50_, only from two (3-acetylaconitine) to six (AC) times. Therefore, they are not suitable to be considered as druggable analgesics. Thus, lappaconitine (LA) shines as a better candidate for analgesics, due to its low toxicity [35, 36]. LA has more or less level of analgesic activity, when comparing to morphine, in different tests on mice and rats [37, 38].

The anti-inflammation action also assists in the analgesic effect in the treatment of some diseases, for example, *Fuzi* alkaloids can mitigate the symptoms of patients with allergic rhinitis [39]. Sneezing, nasal secretions and nasal scratching were alleviated in the study, where the inflammation of nasal mucosal cells was improved through the decrease of histamine content. The use of *Fuzi* was proven to be safe also in clinical application.

*Chuanwu* also has analgesic and anti-inflammatory effect in oedema. In a mice model of carrageenan-induced paw oedema, water extract of *Chuanwu* at 60 mg/kg showed efficient inhibition in oedema [40]. It was believed that mesaconitine (MA), which presented as the highest concentration alkaloids in the sample, responsible for the antinociceptive and anti-inflammatory effects.

### Effects on gynaecological disorders

In TCM practice, *Fuzi* has been used for regulating or balancing the patients’ endocrine system [9]. It could help alleviate many gynaecological symptoms, including abdominal cramps, painful menstruation, irregular menstruation [41], and other gynaecological disorders like climacteric disorder. However, physicians should avoid administering *Aconitum* drugs to pregnant women, as AC has high toxicity to embryo development [42].

## Poisoned symptoms and toxicology

### Chemical aspects of the toxicology of Aconitum

The active compounds found in the *Aconitum* species are mainly alkaloids, with three major groups namely monoester diterpene alkaloids (MDAs), diester diterpene alkaloids (DDAs) and lipoalkaloids. The most important active constituents are C_19_-diterpenoid alkaloids and C20-diterpenoid alkaloids. The compounds are served as a double-edged sword because they are both toxic while having medicinal beneficial effects.

**Fig. 2:**
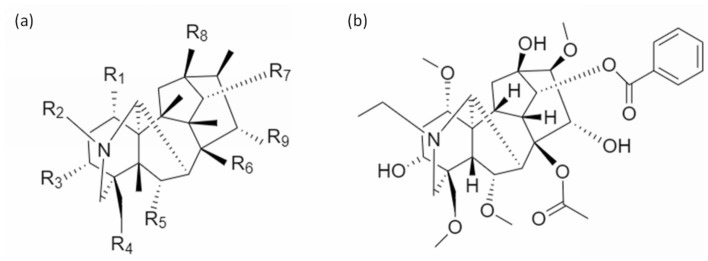
Toxic compounds in *Aconitum.* **a** General chemical skeleton of DDAs. **b** Chemical skeleton of Aconitine

The major toxic compounds are the DDAs including AC, MA, and HA. DDAs are a type of diterpene alkaloids, which shares a C_19_ norditerpenoid skeleton [43, 44]. The skeleton chemical structure is shown in Fig. 2a. The aconites all having the same skeleton, but with different side chains as functional constituents substituted at different sites. Wherever an acetyl group and a benzoyl ester group are attached to C-8 (R_6_ in Fig. 2a) and C-14 (R_7_), the alkaloids would be toxic. AC is a typical example (Fig. 2b). They are the most toxic compounds among all the alkaloids found from the herb. MDA, including benzoylaconine (BAC), benzoylmesaconine (BMA) and benzoylhypaconine (BHA), in comparison, has a lower toxicity level and thus the medicinal value is higher. There is no acetyl substituent in the C-8 (R_6_) in MDAs, which is usually replaced by a hydroxyl group (e.g. BAC, BMA, BHA) [45, 46]. Alkaloids consist of different structures and functional groups will exhibit different activity in the body. From which, compounds with cardiac activities are generalized by Jian et al*.* in 2012 [47]. For C_19_-diterpenoid alkaloids without ester groups, an alpha-hydroxyl group at C-15 (R_9_), a hydroxyl group at C-8 (R_6_), an alpha-methoxyl or hydroxyl group at C-1 (R_1_) and an amine or N-methyl group in ring A could bring possible cardiac activities.

AC is extremely toxic to human, which will cause death with just 2 mg, while the toxic dose is only one-tenth of that [48]. HA, however, is the most potent toxic compound, which exists in a relatively lower proportion. The diterpene alkaloids have either a hydroxyl, acetoxyl or fatty acid acyl group as functional groups, while the effect of many of the compounds is not yet been investigated (Table 1).

**Table 1:** Effective dosages of *Aconitum* herbal extract and compounds

Effect	Herb/Compound	Species	Dose	Ref
*Cardiotonic Effect*				
	Aconitine	Mouse	5.69 ~ 8.49 ug/kg	[119]
	Hypaconitine	Rat	250 ng/mL	[120]
	Higenamine	Rat	5-10 mg/kg	[121]
*Arrhythmic Effect*				
	Aconitine	Cat	20-40ug/kg	[58]
	Aconitine	Rat	1.46 mg/kg	[122]
*Anti-arrhythmic Effect*				
	Hypaconitine	Rat	0.5 mg/kg	[123]
	Lappaconitine	Mouse	1.2–3.8 mg/kg	[124]
*Anti-inflammatory Effect*				
	Aconitine	Human	~ 10 mg/24 h, i.v	[29]
	Mesaconitine	Mouse	0.2–0.5 mg/kg p.o	[51]
	3-acetylaconitine	Mouse	0.18–0.3 mg/kg	[125]
	Lappaconitine	Mouse	6–8 mg/kg p.o	[126]
*Analgesic Effect*				
	Aconitine	Mouse	25 ug/kg	[127]
	Aconitine	Mouse	60 ug/kg	[128]
	Mesaconitine	Mouse	39 ug/kg	[128]
	Hypaconitine	Mouse	10 ug/kg	[128]
	Pyrojesaconitine	Mouse	78 ug/kg	[128]
*Antinociceptive Effect*				
	Aconitine	Mouse	0.028 mg/kg	[34]
	Mesaconitine	Mouse	0.025 mg/kg	[129]
	3-acetylaconitine	Mouse	0.097 mg/kg	[34]
	Lappaconitine	Mouse	2.7 mg/kg	[34]
*Hypoglycemic effect*				
	Aconitan	Mouse	100 mg/kg	[127]
*Anti-oedema Effect*				
	*A. Carmichaeli*	Mouse	60 mg/kg	[40]
*Anti-tumor Effect*				
	*Fuzi* extract	Mouse	360 mg/kg	[130]
	*Aconitum*-derived alkaloids	A172, A549, Hela cell lines	1ug/mL	[131]
		Raji cell line	3ug/mL	[131]

### Clinical symptoms of Aconitum poisoning

Typical symptoms of patients poisoned by aconite derivatives include nausea, vomiting, palpitation, arrhythmia [49], muscle dysfunction, perioral paranesthesia, respiratory tract infection and pain, breathing difficulties, convulsion, gastrointestinal upset [50, 51], and even shock and coma [52, 53]. It is of course lethal in serious cases, which are usually the result of ventricular arrhythmia. There is no direct antidote to aconitine poisoning, only vital supportive measures could be provided to alleviate the situation.

### Toxicology of Aconitum

The toxic diester diterpene alkaloids (DDAs) found in *Aconitum* are well known to affect mainly cardiac function as well as the central nervous system. The DDAs, especially AC, inhibit the inactivation of the voltage-dependent sodium channel by the substitution into a binding site [44, 54]. The binding site is located on the alpha-subunit of the sodium channel protein, which is the specific neurotoxin binding site 2. The sodium channel is thus paralyzed, and the ionic difference, so as the action potential cannot be built up. This leads to the disruption of neurotransmitter release, and thus the neural signal transmitting pathway is disturbed [55]. The nervous system, as well as cardiac and muscular tissue, is observed to be affected mostly and fatally. The toxicity level of some of the compounds isolated from the herbs is listed in Table 2.

**Table 2:** Toxic dose and LD_50_ of the herbs and compounds in *Aconitums*

Herb/Compound	Species	p.o	s.c	i.p	i.v	Ref
Raw *Chuanwu*	Mouse	18,000 mg/kg				[127]
Aconitine	Mouse	1.8 mg/kg	0.24–0.31 mg/kg	0.28–0.34 mg/kg	0.12 mg/kg	[69]
	Mouse		0.55 mg/kg			[128]
	Rat				0.102 mg/kg	[127]
	Rat				0.112 mg/kg	[103]
	Cat				0.07–0.13 mg/kg	
	Dog				0.5 mg/kg	
	Human	2-5 mg				[127]
Mesaconitine	Mouse	1.9 mg/kg	0.19–0.22 mg/kg	0.20–0.23 mg/kg	0.10 mg/kg	[69]
	Mouse		0.25 mg/kg			[128]
	Mouse				0.068 mg/kg	[129]
	Rat				0.158 mg/kg	[103]
Hypaconitine	Mouse	5.8 mg/kg	1.1–1.3 mg/kg	1.0–1.2 mg/kg	0.47 mg/kg	[69]
	Mouse		1.9 mg/kg			[128]
	Rat				0.291 mg/kg	[103]
Benzoylaconitine	Mouse			70 mg/kg	23 mg/kg	[69]
	Rat				> 20.2 mg/kg	[103]
Benzoylmesaconine	Mouse	810 mg/kg	230 mg/kg	240 mg/kg	21 mg/kg	[69]
	Rat				> 18.7 mg/kg	[103]
Benzoylhypaconine	Mouse	830 mg/kg	130 mg/kg	120 mg/kg	23 mg/kg	[69]
	Rat				> 20.4 mg/kg	[103]
Lappaconitine	Mouse				5.9 ~ 11.5 mg/kg	[124]
Jesaconitine	Mouse		0.23 mg/kg			[128]
Songorine	Mouse				106 mg/kg	[129]
Heteratisine	Mouse				147 mg/kg	[129]
Napelline	Mouse				> 147 mg/kg	[129]

Interestingly, aconitine becomes a well-known activator of the sodium channel, and is used to be an experimental means to study the function of the sodium channel in the past decades [56].

#### Toxicology in the cardiac system

Aconite poisoning could be fatal because it affects cardiac function in the way of arrhythmia [57]. Observed symptoms of cardiovascular toxicity include hypotension, chest pain, arrhythmias, palpitation and sinus tachycardia. Due to the effect of DDAs on the sodium channels, their openings are prolonged or disrupted. The calcium and sodium ion exchange through the membrane of the myocardium makes the signalling depolarization and repolarization delayed, which causes arrhythmia. The signals could not be transmitted through the vagus nerve normally, as long as the acetylcholine level could not be controlled at will [58]. The nerves in the cardiac system are affected by prolonging excitation, and thus arrhythmia occurs. Hypotension and bradycardia will occur as a result [59, 60]. Furthermore, the ventromedial nucleus in the hypothalamus could be activated by AC, which will lead to hypotension and bradycardic action [61, 62]. It is also responsible for the slowing down of the circulatory system [63, 64].

AC is sometimes used as an induction agent to arrhythmias in the study of the effect of antiarrhythmic drugs. With different well-studied dosage, AC can create ectopic, tachycardia, fibrillation, *torsades de pointes*, or even death [65].

Noticeably, the above mentioned toxic effects are majorly caused by AC, MA, and HA. Different alkaloids found from the *Aconitum* have the opposite effect, which shows low toxicity but a high medicinal value (will be discussed in the following session).

#### Toxicology in the neural system

The neural system is affected in a similar way as illustrated above [66, 67]. The neural transmitting pathway is disrupted by the AC poisoning, that the neuronal signal cannot normally pass through the synapse. This brings limbs abnormal controls or even paralysis. The DDAs will also cause strong contractions which can result in diarrhoea and abdominal pain [68, 69]. The toxin could also bring damage to the central nervous system, which could lead to temporary or long-term disorder in the brain. In serious case, this can bring shock and coma.

### Clinical aspects of the toxicology of Aconitum

Aconite poisoning has been a problem in the past decades, especially from the ingestion of the *Aconitum* plant. There were more than 41 poisoned cases reported in Hong Kong from 2012 to 2017, and at least 53 patients died because of Aconite poisoning in China [10]. The toxic level of the wild herb is much higher and fatal than the processed herbal drug, therefore the cases in the past were usually very serious, causing deaths [57, 70]. Patients admitted to the hospital suspected of being poisoned by *Aconitum* plants, would show those characteristic symptoms mentioned previously, for example, nausea, vomiting, numbness, hypotension, and extra-systoles or arrhythmia [71, 72]. They would have to be monitored carefully in the intensive care unit with ECG 24-h. In serious case, the patient could result in tetraplegia [73]. Most patients who died of aconite poisoning were due to the collapsed cardiac function, in those cases, the heart beating actions were disrupted irregularly or slowed down [52, 74, 75]. However, with suitable, accurate and on-time treatment, patients who suffered from aconite poisoning could usually be rescued and survived, with or without some consequences [76–78].

## Detoxification of *Aconitum* in traditional and modern ways

### Detoxification by herbal-herbal combinations

Traditional Chinese medicine is particular about syndrome differentiation, and the prescriptions have nearly always more than one drug. The multiple drugs can not only alleviate different symptoms of the patients but also help retaining balance back. Moreover, the drug–drug interaction makes the whole formulation suitable for a single patient personally. Some drugs may be too “hot” to use on their own and should be balanced with some “cool” adjuvants. Picking compatible drugs can not only increase the drug efficacy but also reduce the side effects or even toxicity of the formulation. *Fuzi*, for example, is involved in some common prescription formula, including *Sini Tang (Gancao, ginger, Fuzi)*, as well as *Zhenwu Tang *(*Fuzi, ginger, Chinese peony (Paeonia lactiflora), *etc*.*)*.*

#### Ginseng

*Ginseng (Panax notoginseng)* has the best combination effect with *Aconitum*, in terms of detoxification and enhancing drug action. In vitro experiment has shown that Ginseng could reduce the metabolism of DDAs through inhibition of CYP3A4 expression and activity [79]. In a rat model, *Ginseng* could slow down the metabolisms of DDAs from *Fuzi* when given orally [80]. *Ginseng* combines with *Fuzi* could increase SOD activity and reduce malondialdehyde (MDA), nitrogen oxide and lactate dehydrogenase, which could enhance cardiac cell viability [81]. Through modulating the action of CYP2J3, CYP4A3, and CYP4F11, *ginseng* could reduce the toxicity of *Fuzi and AC* to the heart and cardiac cells [82]. When combines with *Fuzi*, *Ginseng* could reduce the LD_50_ more than Licorice [83].

#### Gancao

*Gancao (Radix glycyrrhiza sp.),* also known as liquorice, is another herb that has been used with the combination of *Aconitum* for hundreds of years. Liquorice extract could induce the function of CYP3A4 and CYP2B6, which can increase the efflux of AC out of the cells via P-glycoprotein [84]. Glycyrrhizin and glycyrrhetinic acid, compounds extracted from *gancao*, has a potent antioxidant effect, which could reduce the cardiac-toxic lipid peroxidation reaction by free radical scavenging [85]. Glycyrrhetinic acid could enhance the anti-apoptotic protein Bcl-2 expression, and reduce pro-apoptotic and pro-inflammatory cytokines such as Fas, Bax, TNF-α and IL-1β, which results in cardiac protection [86]. The combination of *gancao* with *Fuzi* reduce inflammation and ventricular remodelling while enhancing survival in the mice model via TLR4/NF-κB pathway [87].

#### Ganjiang (Dried ginger)

*Ganjiang (Zingiberis rhizoma)* combined with *Fuzi* possess a beneficial therapeutic effect against heart failure by increasing mitochondrial biogenesis via Sirt1, PGC-1α and NRF1 [88]. *Ganjiang* in combined with *Fuzi*, has a better cardiac promotion effect than *Fuzi* alone, by reducing H_2_O_2_ damage and MDA level, while increasing intracellular SOD [89]. The therapeutic effect on heart failure has risen, hemodynamics is enhanced, abnormal activation of neuroendocrine is inhibited [90].

#### Dahuang

*Dahuang (Rhei Radix et Rhizoma)* significantly reduced the DDA levels when combined with *Fuzi* and its derivatives [91]. The side effects of arrhythmia by *Fuzi* such as ventricular premature beats and ventricular tachycardia were alleviated in a dose-dependent manner by *Dahuang*. The effect is increased with the ratio of *Dahuang* to *Fuzi* increases, and the level of toxic alkaloids is reduced [92].

Apart from the above four herbs, *Fangfeng (Saposhnikoviae radix), Huangqi (Astragali radix), Yuanzhi (Polygalae radix), Baishao (Paeoniae radix alba)* also shown increase the LD_50_ and TD_50_ to different extents, when combined with *Fuzi*.

#### Herbs incompatible with Aconitum

In addition to the enhancement of drug effect with reduced toxicity, some herbs are incompatible with *Aconitum*. In TCM, eighteen herbs that are incompatible with each other were arranged in rhythm named “18 incompatible medicaments”. In which, *Beimu (Fritillariae cirrhosae bulbus), Gualou (Trichosanthis fructus), Banxia (Pinelliae rhizome), Bailian (Ampelopsis radix), Baiji (Bletillae rhizome)* were regarded to be incompatible with *Aconitum.* These five herbal drugs were not toxic by themselves. Analysis showed that the cardiotoxicity of *Heishunpian* increases when combined with the five drugs above [93].

### Monitoring toxicity level and quality control

It is found that the herb–herb interaction of *Aconitum* containing formulation could result in significantly different pharmacokinetic fate in vivo [94]*.* This explained why TCM prescriptions usually involve a combination of different drugs and having specific predesigned combinations of drugs that are listed. For example, the use of *Mahuang* and *Fuzi* together could result in a lower alkaloids plasma concentration, but not BAC which is the active compound, when comparing to the use of either one single drug extract [95]. Therefore it is safer and more efficient to use more than one drugs in a specific combination. However, it is worth mentioning that the elimination value and time increased as accumulation may occur during continuous drug intake. It is essential to monitor the patient’s situation before prescribing long term usage of herbal medicine.

Research demonstrated the role of P-glycoprotein (P-gp) which is encoded by the gene Mdr1 in the toxicology of AC [96]. From the experiment, the group of Mdr1a^−/−^ mice showed higher analgesic and toxic effect compared to the normal mice. The level of brain and heart damage, measured by s100-B protein and creatine kinase level, were significantly higher. The histopathological examination also agreed with the result, which the half-life of AC in the knock-down mice was dramatically higher. It is believed that P-gp is involved and responsible for the efflux of AC [97].

Due to the high toxicity level of the *Aconitums,* it is very crucial to have a well-developed and monitored method and system to check the quality of the herbal drugs. The method should cover both qualitative and quantitative aspects well, and help correctly processed and detoxify the medicine [98]. Liquid chromatography is one of the “golden” standards in TCM quality check, and it is again useful in the management of *Aconitum* derived drugs [99]. According to the *Pharmacopoeia of the People’s Republic of China*, moisture content should be less than 15%. By the means of HPLC using isopropanol-ethyl acetate as the solvent, the amount of DDAs should be determined no more than 0.020% in the processed *Fuzi*, and the amount of MDAs should be at least 0.010%. For processed *Chuanwu,* the amount of DDAs should be no more than 0.040%, while MDAs at the range of 0.070 ~ 0.15% [19].

### Detoxification by processing

The toxicity of the drug is greatly reduced even after simple boiling. The amount of AC found in the herbs were lowered after boiling from 10 min to 2 h, with an increasing level of detoxification by the lengthen of time [30, 100, 101]. The process involves mainly the hydrolysis of the DDAs into less toxic monoesters. After 2 h of boiling, the *Baifupian* showed no toxicity to the experimental mice at all, while in the 30 and 60 min group there were measured LD_50_ values. By using HPLC, no AC and very few MA and HA were detected in the 2-h boiling sample. The absence of change in total alkaloid amount found in different boiling time suggested that the toxic compounds were transformed into less toxic derivatives through processing. The experiment also showed that there is no significant difference in the treatment efficacy in different boiling time, suggesting boiling can enhance the safety of consuming the drug while keeping its effect.

Different methods are having different advantages and disadvantages. Boiling is the easiest, but many useful chemicals will be lost by dissolving in water [102]. Cooking or baking could preserve most of the active ingredients, which at least affecting the pharmacological functions. With the advancement of technology, the methods of processing are being re-investigated. Although having very low to undetectable level of DDAs in the *Paofuzhi* samples prepared by method recorded in the *Pharmacopoeia 2015,* the level of MDAs was found to be lower than that of raw *Fuzi* [103, 104]. This is a sacrifice of the function by reducing the toxicity.

A study published recently analyzed the efficacy of detoxication and enhancement of medical effects by different processing methods, *Shengfuzi, Paofuzi,* and four methods inspired by master Zhang Zhongjing, including traditional dry-baked, modern baked, stir-fried, and sand-burnt [104]. The four methods were prepared from crude *Fuzi (Shengfuzi)*, in order to imitate the prescription ideas provided by Zhang. The results showed that the *“Zhifuzi”* produced by the four novel methods had a detectable low and safe amount of DDAs, while dramatically higher amount (three to four times) of MDAs were present. Another study in 2013 had similar results [103]. It suggested that the toxicity level of the processed *Fuzi* have no differences in the steaming or stir-fried herbs with or without saline. Using both methods, the DDAs amount could be greatly reduced by forty to eighty times at the safety level. The difference comes in the functional MDAs part, in which the amounts of MDAs increase were as much as five times in the non-saline-treated *Fuzi*, compared to the insignificant difference in the saline-treated *Fuzi*, as the traditional method. MDAs have a much lower toxicity level, but similar level of efficacy. It was also suggested in the article, that the MDA/DDA ratio should be considered in the quality control of processing *Aconitums.*

And finally, steaming, or autoclaving is the newest technique to detoxify the *Aconitum.* The crude herbs are steamed at 127 °C under 0.15 mPa high pressure. The optimum steaming time should be 60 to 90 min, where toxicity and efficacy could remain balance [105]. They are capable to reduce most of the toxic compounds to less toxic ones, which is the most effective way to make the drug safe to use [106]. The total alkaloids amount and thus the function of the herb do not reduce much. This method is fast and clean, while easy to monitor the quality, as well as cost-effective. It is most commonly used to prepare *Chuanwu* nowadays [107].

It is always crucial to know how a drug or compound being digested and taken into action inside our body. It is also important to keep track of the fate of the substances, pharmacokinetics is the study of this. As a toxic herbal drug, we must learn how efficient our bodies can eliminate the harmful compounds before the drug can be safely used and prescribed. Using the method of ultra-performance liquid chromatography-tandem mass spectrometry (UPLC-MS/MS) [108, 109] or liquid chromatography-electrospray ionization-tandem mass spectrometry (LC–ESI–MS/MS), the alkaloids residue from the drug in the blood serum could be detected and measured fast and accurately.

## Clinical management of *Aconitum*-poisoned cases

As mentioned previously, *Aconitum* poisoning is a serious issue. The consequences are huge for patients suffering from poisons such as AC. This occurs usually by accidentally ingest the wild plant, or unprocessed *Aconitum*, or wrong prescription given by physicians. Lives are threatened because the DDAs are toxic to the heart, neural and gastrointestinal system. When cardiovascular function collapse, the patients are in danger [110].

When the patients suffer from tachyarrhythmia, they would feel dizzy and palpitations occur. It could result in cardiac arrest or myocardial infarction. A 61-year-old man was observed with the above symptoms [111]. The patient was having back pain in the previous 3 months and got prescribed Chinese herbal medicine as analgesics. After consuming a portion of the drug for 1 h, he suffered a lot and admitted to the hospital. He had hypotension, with tachycardia. Lidocaine 100 mg was given immediately, and then with continuous infusion. However, the situation did not improve, and further amiodarone was infused additionally for 36 h. The arrhythmia problem was alleviated and later recovered without other consequences. This was an essential case to prove the usefulness of amiodarone in treating aconitine poisoning induced ventricular tachyarrhythmia. As there is no direct antidote to aconitine poisoning, essential vital support and treatment must be given accurately and effectively. Some other clinical evidence also proved the effect of Flecainide, another antiarrhythmic agent, on treating aconitine poisoning induced ventricular tachyarrhythmia [111].

Another worth mentioning case study was a *Caowu* poisoning that happened on a 48-year-old Chinese man [112]. He consumed some herbal medicinal wine before admitting to the hospital accident and emergency department. He felt chest pain, numbness all over the body, and then dyspnea. Apart from the typical cardiac toxicity, the patient suffered from polycystic renal haemorrhaging as well, due to a family history of kidney disease. The patient was given acticarbon, atropine and intravenous injected lidocaine. The injection lasted for almost 5 h but the prognosis was still poor. Hemoperfusion was carried out instead. The heparin level was lowered to none and half in the two perfusion device respectively. Methylepinephrine was given to maintain blood pressure. The arrhythmia improved around 2 h later, but ventricular bigeminy remained. On the third day after admission to the hospital, the kidney haemorrhage was finally under control. He left the hospital 1 week later. This case emphasizes how important supportive therapy is when dealing with poisoning cases. Blood purification removes the aconitine from the body, and help retaining normal cardiac function. It can also sensitize the heart to antiarrhythmic drugs [77].

A 92-year-old woman took a herbal decoction containing *Fuzi* to treat her mood swings and maintain health, and was soon appeared to be life-threatening with bradycardia and hypotension after 1 h [113]. ECG showed ventricular abnormalities, including sinus bradycardia and low-P wave. The patient was treated with an infusion of saline and inotropic agents for one day, and those symptoms were alleviated and the condition backed to normal. The key to saving patients from aconite poisoning is to find out the poisoned substance as soon as possible, and prescribe with appropriate alleviating agents.

## Discussion

In the past, the ancient Chinese wrote the *materia medica* by trial-and-error. They recorded the usage and pharmacological safe amount and level by experience and tested for the herb-herb combinations one by one until a typical formulation could be concluded. They passed down the methods of processing through generations and improved by years. Case of *Aconitum* poisoning was reported in the modern era as early as 1867 and onwards the toxicity of *Aconitum* has caught more and more attention besides its documented therapeutic effects. A recent study that reviewed 40 cases reporting *Aconitum* poisoning in mainland China from 2004 to 2015 suggested that a majority of the cases appears in persons who believed the medicinally beneficial effect of *Aconitum* in its toxic form [50]. The dosing issue that brings attention to the therapeutic index of *Aconitum* between its pharmacological and toxic limits is therefore critical to the safe use of the herb in clinical practice. A local review from the Hospital Authority of Hong Kong on 52 cases of *Aconitum* poisoning from 2004 to 2009 suggested that overdosing is the major underlying cause of poisoning [114]. There were five recent cases of aconite poisoning from 2016 to 2017, that the patients received medication from the Chinese Medicine Centre of Hospital Authority [115]. They were dispensed with normal doses of processed *Fuzi* but appeared to have symptoms from mild numbness to hypotension. Two of them exhibited bradycardia and were attended in the intensive care unit. They recovered fully after treatment. A report in 2006 recorded the situation that there were 10 cases of aconite poisoning from 2004 to 2006, however, four of them were not prescribed *Aconitum* herbs in the formulation [116]. This is a problem that we must face if there was contamination happened in the herbal medicine, or the quality control failure. In the view of safety control, developing a strict quality control with an indicated range of certain substance in *Aconitum* may be optimal, however, due to the inconsistency of chemical composition between batches and the narrow therapeutic index, the optimal safe range of *Aconitum* substances may be too narrow to be practical. On the contrary, with the aid of modern science, the functions and properties of an herbal drug could be explained and proved by extracts or isolating single compounds. Developing safer methods to detoxify the herbal drugs and enhance drug efficacy in the meantime are beneficial to the society.

Technological advancement has different views on processing methods. The recent studies showed that *Fuzi* processed without soaking in saline have better medicinal effects while keeping the safety level [103, 104]. This is inconsistent with ancient wisdom but could be explained by a few reasons. The safety could not be easily monitored by simple baking of crude herb, as the temperature and condition may not be maintained well in the past. Also, the high salt content in herbal medicine helps increase the storage time of the drug. Finally, the quality check of the drug may be easier, as the appearance of the processed drug would be completely different. The *Pharmacopoeia* should consider introducing or replacing the current method of *Fuzi* processing since the technology level of the pharmaceutical industries has improved a lot.

A single herb could be either an advantageous drug, or a lethal poison at different doses and forms, so it is essential to determine the optimum dosage for a specific disease or case. Traditional Chinese Medicine is inherited for thousands of years, and modern sciences minimize the risk when using herbal medicine. Both are indispensable in the development of pharmacy; the traditional wisdom provides hints and directions to drug development, while modern medicine brings the knowledge to the next level. Herbal-herbal combination to reduce toxicity could be a good point to research deeper into because it requires no additional treatment on a single drug. For example, the alkaloid in *Fangji (Stephania tetrandra)* could inhibit the movement of calcium ions crossing the L-type calcium channel, which in turn lower the toxic effect causing arrhythmic of aconite alkaloids [117]. The addition of the combination is an alternative way to detoxify and increase the safety of toxic herbal drugs, and this is valuable in pharmacological development. On the other hand, different ratios of combination with other drugs could result in different toxicity on *Aconitum* herbs. The five herbs in the “18 incompatible medicaments” were claimed not to be used together with *Aconitum.* However, recent studies reviewed that when combining with different ratios, the toxicity of the combination may not increase [118]. More scientific researches on the topic are needed to illustrate the mechanism of herb-herb combination effects on toxic drugs, not only for *Aconitum*.

## Conclusion

In this review, we update the knowledge advancement about the toxicity and toxicology of *Aconitum* drugs. The drug has been widely used in ethnomedicine especially in China despite its toxicity. It has anti-inflammation, anti-arrhythmic, analgesic and some other effects as a medicine. Increasing evidence has shown the benefits of using *Aconitum* herbs in treatment. However, the risk of cardiotoxicity causing arrhythmic, gastrointestinal upset, or even tetraplegia and death is a serious concern. The poisoned cases were usually caused by inappropriate usage, overdose, long term consumption, or herbal drug of poor quality. Cases are studied and the prescription of western antiarrhythmic drugs such as amiodarone or atropine is an immediate response to save lives. Furthermore, the current methods of *Aconitum* processing are discussed and reviewed. It is concluded that the standard processing method written in the *China Pharmacopoeia* may not be the best one in terms of enhancing the medicinal effect of the drug, and more researches should be done on reflecting the effectiveness. With the correct ratio to combine *Aconitum* with other herbal drugs, such as *Ginseng, Gancao, Ganjiang* and *Dahuang,* toxicity could be reduced and therapeutic effects could be enhanced.

## Data Availability

Not applicable.
